# Supplementary material for: A Pilot Study on the Effects of Transcranial Direct Current Stimulation on Brain Rhythms and Entropy during Self-Paced Finger Movement using the Epoc Helmet
Source: Front Hum Neurosci. 2017 Apr 25;11:201. doi: 10.3389/fnhum.2017.00201 (PMC5408787; doi:10.3389/fnhum.2017.00201)
Supplement: Supplementary file 1 [file Data_Sheet_1.zip › Complementary_results/Band_ratios_Complete_EEG/Gamma_Delta/Gamma-Delta_complete-EEG_Avg AF4-F4-F8.pdf]

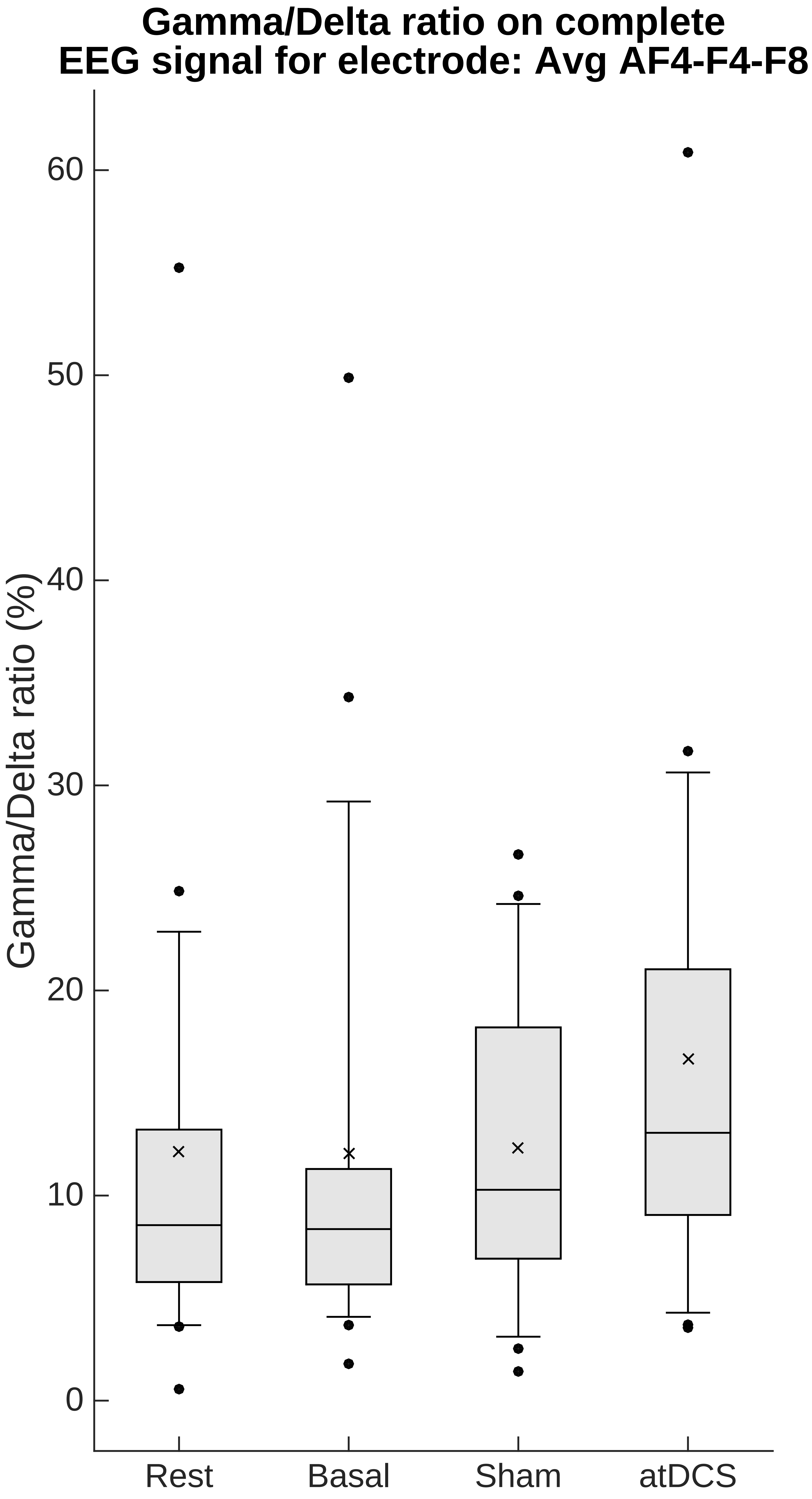

Gamma/Delta ratio on complete
EEG signal for electrode: Avg AF4-F4-F8
Gamma/Delta ratio (%)
0
10
20
30
40
50
60
Rest
Basal
Sham
atDCS
